# ‘’The association of normal tension glaucoma with Buerger’s disease: a case report‘’

**DOI:** 10.1186/1471-2415-14-130

**Published:** 2014-11-14

**Authors:** Yaran Koban, Gorkem Bilgin, Halil Cagatay, Macit Bitargil, Hatice Ozlece, Metin Ekinci, Defne Kalayci

**Affiliations:** Department of Ophthalmology, Kafkas University, Faculty of Medicine, Kars, Turkey; Department of Ophthalmology, Hacettepe University Beytepe Health Center, Ankara, Turkey; Department of Cardiovascular Surgery, Kafkas University, Faculty of Medicine, Kars, Turkey; Department of Neurology, Kafkas University, Faculty of Medicine, Kars, Turkey; Department of Ophthalmology, Ankara Numune Research and Training Hospital, Ankara, Turkey

**Keywords:** Buerger’s disease, Normal tension glaucoma, Branch retinal artery occlusion

## Abstract

**Background:**

To report a case of a 48-year-old man with Buerger’s disease who presented with bilateral normal-tension glaucoma (NTG).

**Case presentation:**

A 48-year-old man who had been diagnosed with Buerger’s disease 12 years ago, and received bilateral below-the-knee amputations for ischemic ulcers of the lower limbs, presented at our clinic due to a sudden loss of visual acuity in the left eye. A fundus exam revealed a cup-to-disc ratio of 0.5 for the right eye and 0.8 for the left eye, arteriolar constriction in both eyes, retinal edema in the inferopapillary area, and splinter hemorrhages and soft exudate in the left eye. We diagnosed the patient as having acute nasal branch retinal artery occlusion in the left eye and bilateral NTG, as a result of the ophthalmologic examination and the other findings.

**Conclusion:**

Although the pathomechanism of NTG is still unknown, previous studies have suggested that patients with NTG show a higher prevalence of vasospastic disorders. We present the second report of NTG associated with Buerger’s disease to be described in the literature.

## Background

Increased intraocular pressure (IOP) is a just one of several risk factors for glaucomatous damage. Nevertheless, the other risk factors such as unstable ocular perfusion may play a more important role in normal-tension glaucoma (NTG) than they do in primary open-angle glaucoma (POAG) with higher IOPs [1, 2]. The term NTG (also called low-tension glaucoma) is used for patients with typical glaucomatous damage of the optic nerve head and visual field loss in spite of normal IOP [3, 4]. Although the pathomechanism of NTG is still unknown, there are some features that may occur more often with this disease: female sex, migraine headache, Raynauld phenomenon, systemic vascular pathologies such as peripheral vasospasm, autoimmune diseases, endothelial dysfunction or coagulopathies [5–7].

Herein, we present a rare case of Buerger’s disease associated with NTG; this is the second reported case of all these pathologies seen together.

## Case presentation

**Figure 1:**
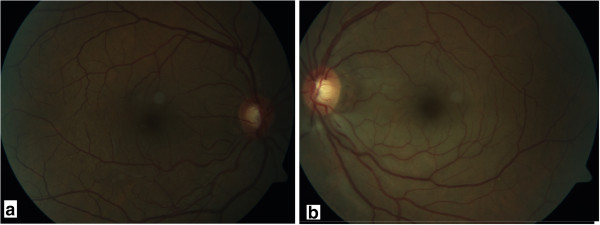
**Fundus photographs of the patients. a**. minimal pallor of the temporal rim and increased cup-to-disc ratio in the right eye. **b**. gross pallor of the rim, retinal thickening in the inferopapillary area, splinter hemorrhages, soft exudates and increased cup-to-disc ratio.

**Figure 2:**
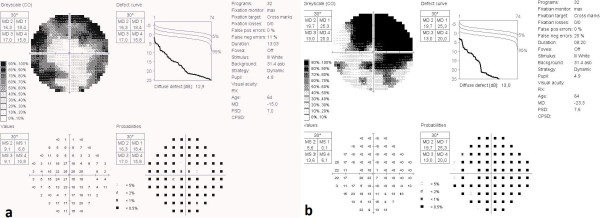
**Visual field of the right and left eyes. a**. Peripheral constriction, decreased retinal sensitivity in the Bjerrum area of the right eye **b**. A small temporal island remaining in the left eye.

**Figure 3:**
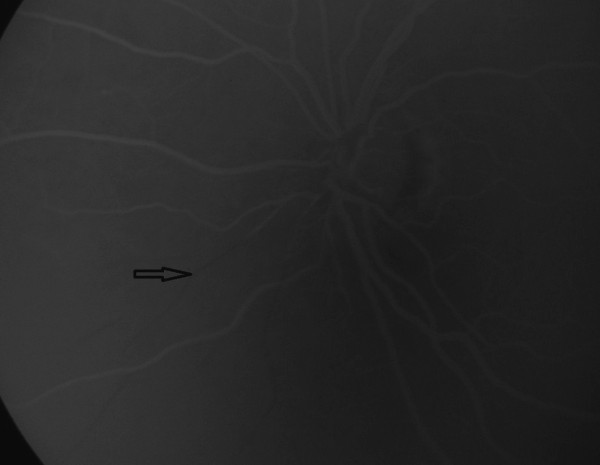
**Later phase fluorescein angiogram shows delayed filling of inferonasal retinal arterioles in the left eye.**

A 48-year-old male patient with acute vision loss in the left eye was admitted to our department. He had been diagnosed with Buerger’s disease 12 years before, and he had undergone bilateral below-the-knee amputations for ischemic ulcers of the lower limbs. He had been taking acetylsalicylic acid 100 mg/day. He also had a 6-year history of diabetes mellitus, controlled by oral antidiabetics. His best-corrected spectacle visual acuity (BCVA) was 20/20 OD and 20/32 OS. Color vision by Ishihara plates was 10/10 in the right eye and 6/10 in the left eye. There was a left relative afferent pupillary defect (RAPD). IOP by standard Goldmann applanation tonometry (Model AT 900, Haag-Streit USA, Mason, OH, USA) was measured as 18 mm Hg OD, 16 mmHg OS. Central corneal thickness was 565 μm OD and 557 μm OS. Gonioscopy revealed bilaterally open iridocorneal angles to the ciliary body band for 360 degrees. Slit-lamp examination of his anterior segments was unremarkable except for a mild posterior subcapsular cataract (P1, according to Lens Opacities Classification System III) in the left eye [8]. Ophtalmic fundus examination revealed a relatively superior entrance to the central retinal artery, temporal peripapillary atrophy, arteriolar constriction in both eyes, retinal edema in the inferopapillary area, splinter hemorrhages, and soft exudate in the left eye. The cup-to-disc ratio was 0.5 in the right eye, and 0.8 in the left (Figure 1). Visual field test showed peripheral constriction, decreased retinal sensitivity in the Bjerrum area of the right eye, and a small temporal island remaining in the left eye (Figure 2). Fundus fluorescein angiography revealed a delay in inferonasal branch retinal artery filling in the left eye (Figure 3). Bilateral carotid artery color doppler ultrasound and cranial magnetic resonance imaging showed left carotid artery stenosis associated with thrombosis. We diagnosed the patient as having acute inferonasal branch retinal artery occlusion (BRAO) in the left eye and bilateral NTG, as a result of the ophthalmologic examination and the other findings.

## Conclusion

Buerger’s disease, also known as thromboangiitis obliterans, is a segmental nonatherosclerotic inflammatory disorder that primarily involves the small and medium arteries, veins and nerves of the extremities, and is observed in young and middle-aged male smokers. It is a rare vasculitis characterized by a highly cellular inflammatory thrombus with relative sparing of the vessel wall and can be distinguished from other types of vasculitis based on the rarity of systemic signs and symptoms and the absence of both elevated acute-phase reactants and immunological markers. Although smoking, genetics, hypercoagulability, endothelial dysfunction, infection and immunologic mechanisms have been proposed as factors influencing the onset of Buerger’s disease, the true cause of Buerger’s disease is still unknown [9, 10].

It is typically a disease affecting vessels in distal parts of the extremities, but it has also been reported to involve unusual vascular beds, the great vessels, and pulmonary, proximal extremity, mesenteric, cerebral, coronary, renal, pelvic, and ophthalmic arteries [11].

The ophthalmic artery (OA) is the main blood supply to the orbit and gives rise to ciliary arteries that supply the choroid and optic nerve head, and to the central retinal artery that supplies the retina [12]. Early sclerotic changes and arteriosclerosis of the retinal arteries were previously reported in Buerger’s disease, suggesting that changes in blood flow by vasospasm and thrombotic occlusions ocur within the ocular arteries [9, 13]. However, except for our case, there is only one case in the literature that describes Buerger’s disease with dysfunctional regulation of ocular blood flow within the optic nerve head and retinal arteriole having caused NTG [13].

NTG is a major subtype of glaucoma, associated with IOP, which is within the statistically normal range of the population. The etiology and pathogenesis of NTG remains unclear; however, genetics, autoimmune mechanisms and vasospasm have been suggested as possible major etiologic factors that cause local ocular blood flow disturbance within the optic nerve head [14–16]. Several authors have demonstrated a difference in resting pulsatile ocular blood flow (POBF) in relation to IOP, with a lower POBF in those with a higher IOP [17, 18]. Quaranta *et al*. demonstrated a lower POBF in NTG patients when compared with healthy controls despite similar resting IOP and suggested these findings may be consistent with a lack of myogenic autoregulation in response to IOP induced modifications of the perfusion pressure in these patients [19].

Bilateral NTG was diagnosed in our patient with IOP consistently lower than 21 mmHg on repeated measurements, large cup-to-disc ratio and visual field defects. The limitation of the sudy is that IOP assessment was not performed by a 24 hour phasing. Quaranta *et al*. showed that the IOP across three daytime points (1000, 1400, and 1800 hours) was greater (23.6 mmHg) compared with the pressure measured across the three nighttime points (2200, 0200, and 0600 hours) (21.5 mmHg). Also the mean sitting IOP recorded at 1000, 2200, 0200, and 0600 hours was significantly lower than that measured in the supine position [20].

Ours is the second case in the literature presenting the association of Buerger’s disease, retinal artery occlusion and NTG. Decreased cerebral blood flow has also been associated with glaucomatous damage and visual field deficits. (12) Doppler imaging has demonstrated increased blood flow resistance and decreased blood flow velocities in the OA of patients with NTG, thus suggesting the role that changes in blood supply can play and/or correlate with in optic nerve head pathology [21]. Thus, in our case, left carotid artery stenosis associated with thrombosis accompanied by Buerger’s disease is another risk factor causing retinal artery occlusion and NTG in the same eye. While bilateral arteriolar constriction, optic nerve head changes and visual field defects may have been caused by vasospasm and local ocular blood flow disturbance due to Buerger’s disease, advanced glaucomatous damage in the left eye (while the right eye had moderate damage) and BRAO may have been caused by the additive effect of left carotid artery stenosis.

Buerger’s disease, which causes systemic blood flow disturbance, is a systemic disease. Because the disease typically presents in patients <45 years of age, vascular and neurological defects that may lead to vision loss may occur at a high probability, depending on the dysfunctional regulation of ocular blood flow over time. Therefore, Buerger’s disease should be considered as an etiology of BRAO and NTG and regular ophthalmologic examination should be included in follow-up.

### Consent

Written informed consent was obtained from the patient for publication of this case report and any accompanying images.

## Abbreviations

NTG: Normal-tension glaucoma
IOP: Intraocular pressure
POAG: Primary open-angle glaucoma
BRAO: Branch retinal artery occlusion
OD: Right eye
OS: Left eye
OA: Ophthalmic artery.
